# Effectiveness of Chinese massage therapy (*Tui Na*) for chronic low back pain: study protocol for a randomized controlled trial

**DOI:** 10.1186/1745-6215-15-418

**Published:** 2014-10-29

**Authors:** Mingxiao Yang, Yue Feng, Hong Pei, Shufang Deng, Minyu Wang, Xianjun Xiao, Hui Zheng, Zhenhong Lai, Jiao Chen, Xiang Li, Xiaoguo He, Fanrong Liang

**Affiliations:** Chengdu University of Traditional Chinese Medicine, No.37, Shierqiao Road, Jinniu District, Chengdu, Sichuan 610072 China; Tuina Massage Department, The Fifth Hospital of Sichuan Province, No. 66, Shangye District, Chengdu, Sichuan 610031 China

**Keywords:** Chronic low back pain, Effectiveness, Randomized controlled trial, *Tuina*, Chinese massage therapy

## Abstract

**Background:**

Low back pain is a common, disabling musculoskeletal disorder in both developing and developed countries. Although often recommended, the potential efficacy of massage therapy in general, and Chinese massage (*tuina*) in particular, for relief of chronic low back pain (CLBP) has not been fully established due to inadequate sample sizes, low methodological quality, and subclinical dosing regimens of trials to date. Thus, the purpose of this randomized controlled trial (RCT) is to evaluate the comparative effectiveness of *tuina* massage therapy versus conventional analgesics for CLBP.

**Methods/Design:**

The present study is a single center, two-arm, open-label RCT. A total of 150 eligible CLBP patients will be randomly assigned to either a *tuina* treatment group or a conventional drug control group in a 1:1 ratio. Patients in the *tuina* group receive a 20 minutes, 4-step treatment protocol which includes both structural and relaxation massage, administered in 20 sessions over a period of 4 weeks. Patients in the conventional drug control group are instructed to take a specific daily dose of ibuprofen. The primary outcome measure is the change from baseline back pain and function, measured by Roland-Morris Disability Questionnaire, at two months. Secondary outcome measures include the visual analogue scale, Japanese orthopedic association score (JOAS), and McGill pain questionnaire.

**Discussion:**

The design and methodological rigor of this trial will allow for collection of valuable data to evaluate the efficacy of a specific *tuina* protocol for treating CLBP. This trial will therefore contribute to providing a solid foundation for clinical treatment of CLBP, as well as future research in massage therapy.

**Trial registration:**

This trial was registered with ClinicalTrials.gov of the National Institute of Health on 22 October 2013 (http://NCT01973010).

## Background

Low back pain is a common, disabling musculoskeletal disorder in both developing and developed countries [1], with chronic low back pain (CLBP) being the leading cause of disability and absenteeism, worldwide [2]. The lifetime prevalence of low back pain in the general population is estimated to be between 70 and 85%, with an annual incidence rate ranging from 6.3 to 15.4% [1, 3]. According to the 2000 UK guidelines, 90% of episodes of acute low back pain resolve spontaneously, with patients returning to work within a month [4, 5]. However, several studies claim that low back pain and related loss of function persist for 3 to 12 months [6, 7] with more than 25% of patients experiencing recurrence of low back pain within a year [8]. Up to 7% of patients develop CLBP [9]. Because CLBP is not life-threatening, its enormous social and economic cost is often underestimated. As of 2010, the global burden of CLBP was reported to be comparable to that of cardiovascular disease, infectious disease, and cancer [10–12].

The pathophysiology of CLBP is poorly understood due to difficulties in localizing the source of the pain [13]. Potential causes of low back pain include, but are not limited to, changes in the spinal disc structure with aging and degeneration, as well as changes in local concentrations of cytokines such as matrix metalloproteinases, phospholipase A2, nitric oxide, and tumor necrosis factor-α [14]. Based on data from the Low Back Pain Group of the Bone and Joint Health Strategies for Europe Project, most cases of low back pain are non-specific, with a specific cause being identified in only 10% of cases; non-specific low back pain is, by its definition, a symptom of unknown cause [15]. Specific conditions contributing to low back pain include degeneration, inflammation, infective and neoplastic causes, metabolic bone diseases, referred pain, psychogenic pain, trauma, and congenital disorders [16].

In clinical practice, a focused medical history and comprehensive physical examination are required for doctors to make appropriate treatment recommendations. Diagnostic tests, including imaging studies such as X-ray, magnetic resonance imaging (MRI), and computed tomography (CT) are not routinely recommended for uncomplicated low back pain, except when severe or progressive deficits are present or when serious potential factors are suspected [17]. Conventional medications in several classes have been shown to have moderate short-term benefits for patients with low back pain. For most patients, first-line medication options include analgesics like acetaminophen or nonsteroidal anti-inflammatory drugs (NSAIDs) [18, 19]. These medications have limited effectiveness and are frequently associated with undesirable side-effects on gastrointestinal, renovascular, and other systems [20–22]. The heavy economic burden of low back pain has a huge impact on individuals, families, communities, governments, and businesses throughout the world. Thus, alternative back pain treatments are needed that minimize cost and maximize health benefit [23]. Although non-pharmacological treatments such as bed rest, exercise, acupuncture, massage, spinal manipulation, yoga, and cognitive behavioral therapy are commonly prescribed in addition to pharmacologic therapy, the evidence supporting their efficacy is inconclusive.

Chinese massage therapy (referred to as *tuina*) is commonly defined as the ancient healing art of fingers and strength [24]. *Tuina* has been practiced in China for over 5000 years [25]. It is a well-respected treatment modality known to be helpful and safe for a wide range of conditions. For these reasons, it is rapidly gaining international favor [26]. *Tuina* involves a wide range of technical manipulations conducted by a practitioner’s finger, hand, elbow, knee, or foot applied to muscle or soft tissue at specific body locations. It incorporates many of the principles of acupuncture including the use of acupoints. For instance, *tuina* often uses manual techniques such as pushing, rubbing, kneading, or high-intensity, high-frequency patting to clear energy blocks along specific meridians associated with particular conditions [24].

At present, Chinese massage therapy is widely accepted as a complementary and alternative medicine modality [27]. Its efficacy has been demonstrated for the management of many medical and psychiatric conditions. These include, but are not limited to, failure to thrive in preterm infants, major depressive disorder, substance abuse and dependence, pain syndromes, and immune and autoimmune conditions [28–31]. Massage therapy has been shown to be particularly effective for disorders of musculoskeletal origin [32]. However, due to a paucity of high-quality studies, there remains controversy about the efficacy and effectiveness of massage. Many clinical trials suffer from inadequate sample size, low methodological quality, and/or sub-therapeutic massage dosing [33]. As a result, the findings of recent systematic reviews about massage therapy for low back pain are consistently inconclusive, due to the methodology flaws in the primary studies they reference. Therefore, studies without these flaws are important to confirm the efficacy and effectiveness of *tuina* for low back pain [32, 34]. This trial will therefore contribute to providing a solid foundation for clinical treatment of CLBP, as well as future research in massage therapy.

## Methods/Design

### Ethics approval

All trial procedures place the participant’s benefit as the highest priority. The present study protocol has already been ethically reviewed and approved by the Sichuan Regional Ethics Review Committee on Traditional Chinese Medicine (TCM) with the ethical approval identifier 2013KL-002.

### Study design

**Figure 1:**
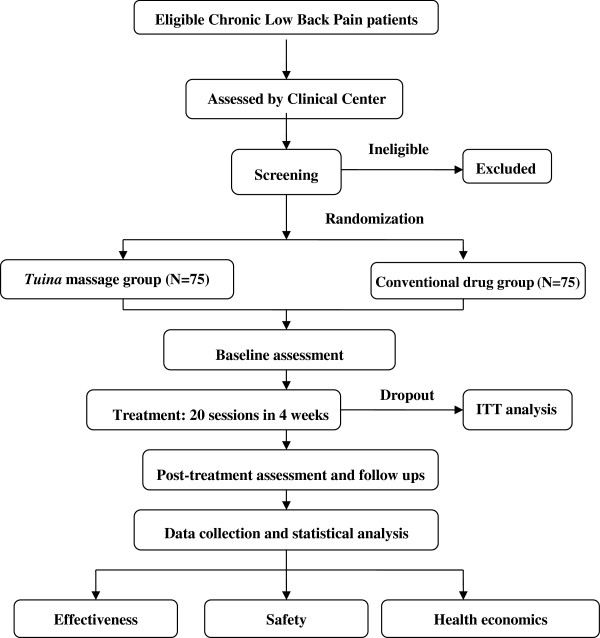
**Trial flow chart.** The present study is a single center, randomized, conventional drug controlled, open-labeled trial. A total of 150 eligible CLBP patients are anticipated to be included and randomly allocated to either *tuina* massage treatment group or conventional drug control group, in a 1:1 ratio. Patients in the *tuina* group receive a four-step massage treatment consisting both structural and relaxation massage. *Tuina* massage treatment consists of 20 sessions of approximately 20 minutes duration, each administered over a period of four weeks. Patients in the conventional drug control group are instructed to administer ibuprofen. The effectiveness, safety, and health economics of *tuina* massage versus conventional drugs is analyzed after data collection. The full analysis set including the dropout will be analyzed by the intention-to-treat (ITT) population analysis.

The present study is a single center, two-arm, open-label randomized controlled trial. All trial procedures will be conducted in the Fifth Hospital of Sichuan Province, Chengdu, China. A total of 150 eligible CLBP patients will be randomly assigned to either a *tuina* treatment group or a conventional drug control group in a 1:1 ratio (Figure 1).

### Participants

Eligible participants include patients diagnosed with CLBP according to the clinical guideline for traditional Chinese medicine (the Criteria of Diagnosis and Therapeutic Effect of Diseases and Syndromes in Traditional Chinese Medicine, ZY/T001.1-94) [35]. Patients presenting for the first time to either the Neurology Department or the *Tuina* Department in the hospital for low back pain for over three months (without relief for over two weeks) will be informed of this trial. If the patient expresses interest, a clinical trial communicator will contact them to provide a brief introduction about the trial. Detailed information regarding patients’ low back pain will be acquired for further assessment of inclusion eligibility according to the following criteria.

#### Inclusion criteria

In order to be included in this trial, participants have to be: (i) diagnosed with CLBP according to the aforementioned diagnostic methods; (ii) between 20 to 55-year-old; (iii) free of immune dysfunction, genetic disorders, or severe cardiovascular diseases; (iv) free from prolapse of the central lumbar intervertebral disc, ankylosing spondylitis, spinal stenosis, intraspinal tumor, and tuberculosis; (v) free from any kind of hormonal or anti-rheumatic drugs for the two months prior to inclusion; (vi) free from allergies to hormones or analgesics; and (vii) able to understand and accept all trial procedures and cooperate with clinical physicians’ practices.

#### Exclusion criteria

Participants with any one of the following conditions will be excluded from this trial: (i) pregnancy; (ii) severe heart, liver, or renal dysfunction; (iii) tumor; (iv) any hematological, respiratory, or cardiovascular disease; (v) any psychiatric disorder; (vi) severe nervous dysfunction resulting from vertebral pulp prolapse, (vii) cauda equina compression or other indications for spinal surgery; (viii) sciatica, (ix) lumbar surgery within the past three years; (x) any disorders that may confound the assessment of *tuina* efficacy, such as severe fibromyalgia or rheumatic arthritis; (xi) ongoing corticosteroid or NSAID use, (xii) history of allergy to NSAIDs; and (xiii) have received *tuina* therapy for CLBP in the past three months.

Benefits and risks are clearly explained to eligible patients before inclusion. Prior to trial inclusion, all eligible patients will provide their written consent.

### Randomization

In this trial, participants will be randomly assigned to either the *tuina* group or the ibuprofen group in a 1:1 ratio using a random number generator (SPSS 16.0, SPSS Inc, Chicago, IL, USA).

### Blinding

As an open-label clinical trial, both patients and clinicians know which treatment approach they will receive, and they are required to cooperate with their physicians or therapists prior to treatment. The assessment of clinical efficacy will be performed over the telephone by a clinical assessor who will be masked to the treatment assignment. During the data collection and analysis stages, the clinical researcher, assessor, and statistician do not share study information with each other.

### Interventions

The *tuina* protocol used in this trial is the same as those used in our previous studies [36, 37]. It includes both relaxation methods and structural methods such as rolling, pressing, jostling, friction, pulling, and tapping.

#### Tuina treatment group

The CLBP patient will receive *tuina* massage therapy for 20 minutes, five times a week for a total of four weeks. Lumbar function will be assessed at baseline as well as at four, six, and eight weeks after the baseline assessment.

In this arm of the study, the *tuina* therapist will administer a four-step protocol intended to ease low back pain and improve lumbar function by promoting *Qi* movement (which according to traditional Chinese medicine theory activates blood circulation), or by inducing a state of general relaxation sensation while addressing specific structural issues determined by the clinician to be likely to contribute to the patient’s CLBP. The specific protocol used is described below.

##### Step one: relaxation manipulation

Patients are instructed by the *tuina* therapist to lie in the prone position and to relax their mind and body. Low back pain conditions can be carefully examined by postural and palpatory assessment prior to treatment. Tender tissues, trigger points, contracted muscle tissue (knots), and nodules are identified for further treatment. The therapist will use his forearm to gently roll on the low back area from the bilateral erector spinae muscles to both thighs, and then continuously from the low back to the gastrocnemius muscle through to the buttocks, for a total of five minutes. During this time, the force and pressure are gradually increased with the intention of smoothing the *Qi* pathways to promote *Qi* movement in different physiological layers. Then, the therapist will apply mild force and pressure with overlapped palms to the lumbosacral area and lower limbs for five minutes, moving inferiorly and concluding with the gastrocnemius muscle. This technique will be performed to resolve adhesions and increase general circulation.

##### Step two: local pressing pain point manipulation

The pressing pain point, or namely *A-Shi* point in acupuncture theory, is the tender local dermal or muscular area. It is generally recognized as reflecting the underlying condition and is frequently manipulated to stop pain. In this step, the therapist will apply muscle pressing, stripping, and deep tissue kneading to the pressing pain point in the lumbar region in a direction perpendicular to the erector spinae. The pressure and amplitude shall be gradually intensified and enlarged throughout the five-minute manipulation, which is intended to unblock *Qi* stagnation, remove blood stasis by separating adherent fascicles, and resolve contracted nodules of muscle. The amount of force used is determined by the patient’s *Deqi* sensation, often described as a dull pain, heaviness, numbness, or soreness, and commonly regarded as an indicator of manipulation effectiveness in acupuncture and *tuina* [38–40].

##### Step three: lumbar structural rectification

Lumbar structural rectification is performed after the above two procedures have relieved the tensions of muscles and soft tissues. The patient will be instructed to lie on his or her side (with the affected side up). The affected leg is slightly flexed at the hip and knee in a relaxed position, while the other leg is naturally extended on the massage table. The therapist stands facing the patient with two hands joined and elbows bent. One of the therapist’s elbows will be fixed on the anterior aspect of the patient’s shoulder, while the other will be placed in the posterior-lateral aspect of the patient’s iliac bone, in the gluteal area of the external hip rotators.

First, the therapist can exert a gentle torque to align the patient’s lower back and perform a slight shake to relax the area. Second, the therapist will push down (toward the table) and stretch the patient’s shoulder anteriorly while stretching the hips posteriorly, rotating the lumbar vertebra along the spinal axis to release the fixing points instantly. After the lumbar muscles are sufficiently relaxed by gentle tractions and twisting forces, the therapist can twist the lumbar muscles slightly further to remove any remaining slack in them. The therapist shall hold this position for a moment and then made an abrupt pulling motion to advance the stretch by 5 to 10 degrees.

##### Step four: tapping manipulation

The therapist use his or her palm to tap the lumbosacral area for two minutes to generate a warm sensation in deep tissue, and then rub the area superficial to the back pain, as well as the bilateral lines of the urinary bladder meridian.

#### Conventional control group

Patients in the control group will receive a conventional pharmacological treatment regimen of one 0.3 g capsule of sustained-release ibuprofen, taken three times each day (Ibuprofen Sustained Release Capsules, 0.3 g per capsule, Sino-GlaxoSmithKline, Tianjin, China).

### Study therapists

All practitioners in this trial are licensed TCM *tuina* therapists with at least five years clinical experience in the hospital’s *Tuina* Department. Before taking part in this trial, they will be required to complete a 40-hour training course to master the study protocol. When completed, clinicians will be required to pass an examination during which they are asked to recite the protocol verbally and provide a demonstration of each technique.

### Outcome measurements

The efficacy of massage therapy for the treatment of CLBP is assessed by the primary outcome measure: change in back pain and function from baseline as measured by the Roland-Morris Disability Questionnaire at four time points (baseline, four, six, and eight weeks). Secondary outcome measures also measured at these four time points included the (i) 100-point visual analog scale (VAS), (ii) Japanese Orthopedic Association Score to assess the improvement of back function, and (iii) McGill pain questionnaire to assess the alleviation of pain. Table 1 demonstrates all measurements and measuring time points.

**Table 1:** **Trial process chart**

Period	Inclusion	Treatment	Follow-up	
Assessment	Baseline	First	Second	Third
**Measure point**	0 weeks after inclusion	4 weeks after inclusion	6 weeks after inclusion	8 weeks after inclusion
**Diagnosis and treatment**				
Inclusion confirmed	√			
Informed consent	√			
Body sign	√	√		
Disease history	√			
Treatment history	√			
Comorbidity	√	√	√	√
Current treatment	√	√	√	√
**Pain condition and lumbar function assessment**				
Visual Analogue Scale	√	√	√	√
Roland-Morris Disability Questionnaire	√	√	√	√
McGill pain questionnaire	√	√	√	√
Japanese Orthopedic Association Score	√	√	√	√
**Data collection and statistical analysis**				
Adverse event		√		
Causes of dropout		√	√	√
Safety analysis		√		
Compliance analysis		√	√	√
Health economics		√		

### Safety

Therapeutic safety will be monitored by assessment of patient symptoms as well as blood, urine, and stool tests conducted pre- and post-treatment. Adverse events such as changes in pain, syncope, vertigo, and lumbar function degradation, will be carefully recorded in the case report form.

### Health economics

All costs associated with this trial will be recorded. They primarily include the medical costs for direct treatment of the CLBP, such as inpatient bed fees, medication fees, massage treatment fees, usual care, and testing fees. Additionally, any cost of treatment for adverse events will be recorded and included in the health economics evaluation.

### Sample size calculation

Sample size was calculated by G*Power 3 software, developed by the Institute for Experimental Psychology (Heinrich-Heine University, Germany). For this trial, it was determined prospectively that α =0.05 and 1-β =0.90. Consistent with a previous trial on massage for lumbar disc herniation [41], a total of 150 participants will be included in this trial (75 in each group) to compensate for an anticipated dropout rate of 15%.

### Data analysis

Demographic and baseline data will be analyzed with standard, descriptive statistics. Between-group differences will be tested using repeated measure analyses of variance. The accepted level of significance for all analyses was *P* <0.05. The whole data analysis process will be performed by statisticians who are independent from the research team and blinded to the group settings. SPSS software (SPSS 12.0 KO for Microsoft Windows® SPSS, Inc., Chicago, IL, USA ) was used to perform the data analysis.

## Discussion

The present trial is a comparative effectiveness study of TCM *tuina* massage and conventional analgesics for pain relief and function recovery in patients with CLBP. The massage techniques used in this trial combine relaxation and structural massage methods applied in a manner which is consistent with TCM theory and is based on recognition of the same energetic meridians and acupoints used in acupuncture.

According to TCM, a state of health reflects an underlying state of balance in the *Qi* and blood of the human body. Pain is usually caused by obstruction of *Qi* and consequently of blood circulation in the affected body region. Pathogenic factors such as blood stasis, *Qi* stagnation, phlegm, dampness, and others can be identified as causative factors in the blockage. Thus, the central therapeutic goal of *tuina* is to remove energetic blocks which lead to *Qi* stagnation. This leads to increased circulation and reduction of localized edema, which helps to reduce associated pain.

The *A-Shi* point in TCM is the site on the body surface which reproduces the specific pain being treated when it is gently pressed. Its location indicates the precise place where *Qi* and blood are blocked. Manipulation at the *A-Shi* point is done with the intention of removing the energetic block there to promote the free movement of *Qi* and improve blood circulation in the region. Studies have demonstrated that one mechanism by which massage therapy appears to be clinically beneficial is by reducing inflammation and promoting mitochondrial biogenesis for repair of damaged skeletal muscle [42].

A recent trial reported no clinically meaningful difference in the effectiveness of structural and relaxation massage [43]. In contrast, this trial compares the efficacy of a specific form of massage based on the principles of TCM (*tuina*) with conventional analgesics (ibuprofen) for pain relief and functional recovery in patients with CLBP. Each of these types of massage is done with very different intentions underlying theoretical frameworks. Therefore, it is important to develop a degree of specificity in referring to a type of massage, both in research and in prescribing clinical massage for a particular condition. There is no placebo control in this trial because of the difficulties in designing a proper placebo for massage therapy which have been described by others [44]. A possible limitation of this study is that it may possibly be difficult to maintain high compliance in follow-up, due to the long interval since the completion of trial. Proper actions, such as frequent telephone interview, will be taken to improve compliance.

The design and methodological rigor of this trial will allow for collection of valuable, high-quality data to evaluate the efficacy of a specific *tuina* protocol for treating CLBP, and so will contribute to providing a solid foundation for the clinical treatment of CLBP, as well as future research in massage therapy.

## Trial status

This trial is recruiting patients now. Participant recruitment started in June 2013, and is expected to end in December 2014.

## Abbreviations

CLBP: Chronic Low Back Pain
CT: Computed tomography
JOAS: Japanese Orthopedic Association Score
MRI: Magnetic resonance imaging
NSAIDs: Nonsteroidal anti-inflammatory drugs
TCM: Traditional Chinese Medicine
VAS: Visual Analogue Scale
ITT: Intention-to-Treat.
